# Successful Coronary Protection during TAVI in Heavily Calcified Aortic Leaflets in Patient with Short and Low Left Coronary System

**DOI:** 10.1155/2018/2758170

**Published:** 2018-05-14

**Authors:** Mohamad Kabach, Abdulah Alrifai, Lawrence Lovitz, Mark Rothenberg, Cristiano Faber, Marcos Nores

**Affiliations:** JFK Medical Center, University of Miami School of Medicine, Atlantis, FL, USA

## Abstract

Transcatheter aortic valve replacement has been recently approved for patients who are high or intermediate risk for surgical aortic valve replacement. The procedure is associated with several known complications including coronary related complications. Coronary obstruction is rare but disastrous complication, and it is associated with a high mortality rate. Coronary protection technique has emerged as a preemptive technique to avoid this complication. We present a case of successful coronary protection during TAVR in severely calcified left cusp in patient with short and low left ostium.

## 1. Introduction

Surgical aortic valve replacement has been the mainstay of treatment of symptomatic severe aortic stenosis. The transcatheter approach comes to the rescue as a less invasive treatment in these high-risk patients [1] or old patients with intermediate risk [2] as well as inoperable patients [3]. TAVR has been associated with vascular, cerebrovascular, valvular, and conduction complications. A rare, life-threatening complication of TAVR is a coronary ostial obstruction. Several cases were reported in this field [4]. A systematic review of reported cases suggests that it occurs more often in women and in patients receiving a balloon-expandable valve. However, this complication has not been evaluated in comparative studies of CoreValve and SAPIEN transcatheter valve models [5]. Coronary protection during TAVI is a preemptive technique recommended in certain cases to avoid this complication. We report a case of successful coronary protection in patient with severely calcified aortic valve in patient with short and low left coronary system.

## 2. Case Presentation

**Figure 1:**
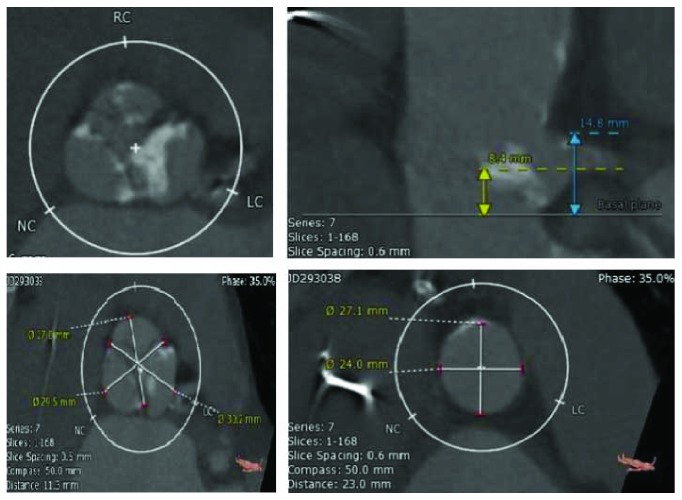
Cardiac computed tomography angiography showed severely calcified aortic leaflets with short and low left coronary system with coronary ostial height of 8.4 mm.

**Figure 2:**
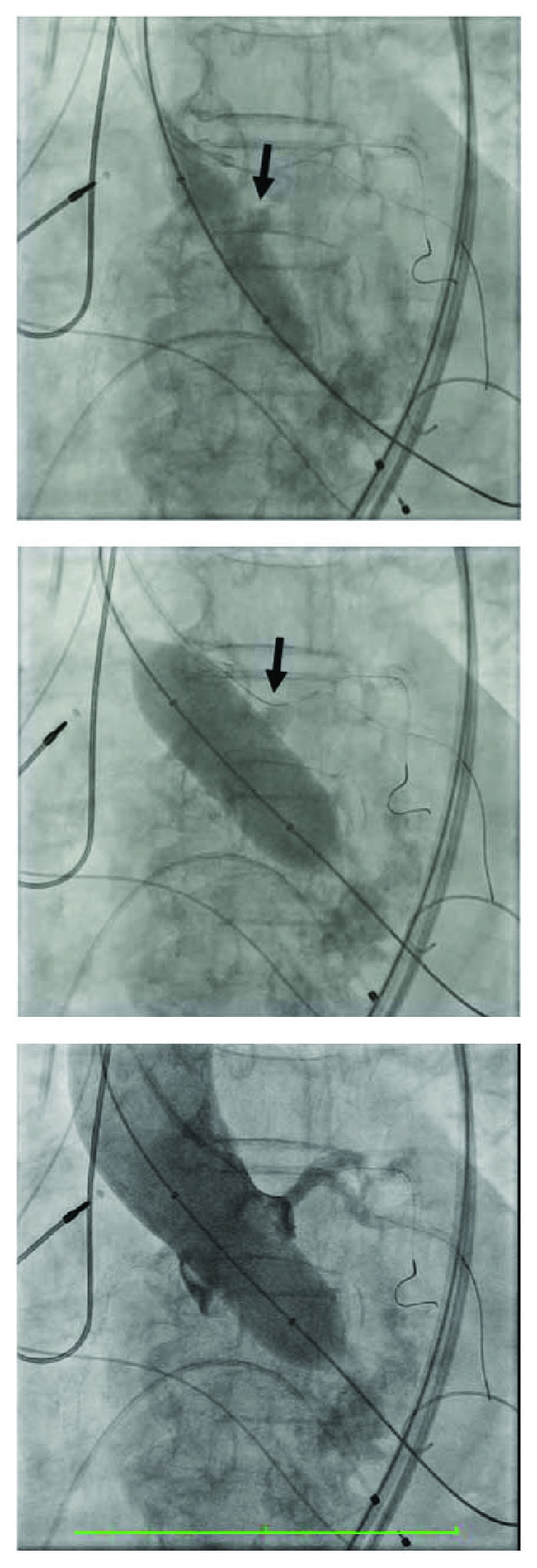
Balloon aortic valvuloplasty, notice the deformed wires and the large piece of calcium in the left cusp moved right over the left main coronary artery (arrows). Simultaneous aortic root injection showing decreased coronary flow TIMI-2. Echocardiogram demonstrated severe aortic stenosis with mean aortic valve pressure gradient of 68.6 mmHg and peak velocity of 5.15 m/s.

**Figure 3:**
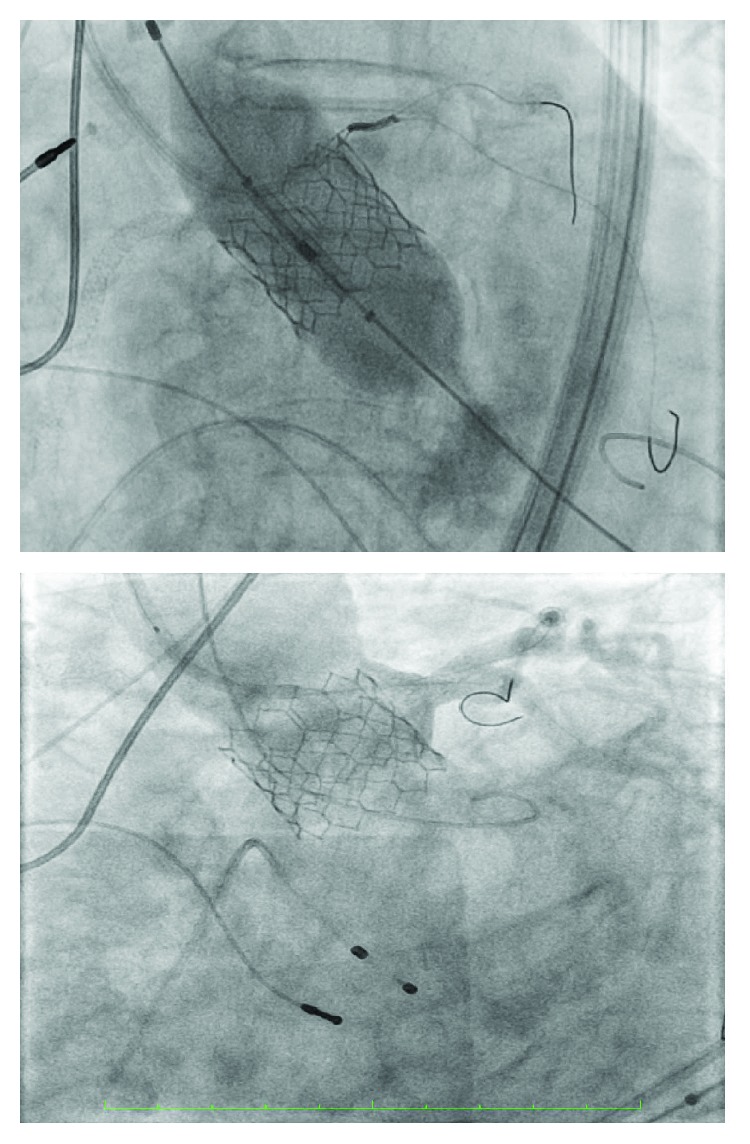
Final pictures demonstrating implantation of the Edward S3 valve with the kissing stents deflated prior and the final results after final implantation.

An 86-year-old woman with known history of coronary artery disease and sick sinus syndrome was admitted to the hospital with dyspnea, orthopnea, and exertional dizziness. Physical exam revealed bibasilar crackles, a grade 5 crescendo-decrescendo murmur, elevated JVP, and lower extremity edema. Laboratory findings were pertinent for creatinine of 1.73, BUN of 45, and NT-pro-BNP of 10k. The rest of her physical exam and laboratory was normal. Electrocardiogram showed normal sinus rhythm with known left bundle branch block. 2D echocardiogram demonstrated severe aortic stenosis with mean aortic valve pressure gradient of 68.6 mmHg and peak velocity of 5.15 m/s with preserved systolic function. She received intravenous diuretics with some clinical improvement. She was seen and evaluated by cardiothoracic surgeon for evaluation of aortic valve replacement but deemed high risk for surgical aortic valve replacement with an estimated surgical mortality by Society of Thoracic Surgeons score of 8.1%. Cardiac computed tomography angiography was done as part of transcatheter aortic valve replacement evaluation and showed severely calcified aortic leaflets with short and low left coronary system with coronary ostial height of 8.4 mm (Figure 1). Special attention was paid to the left coronary leaflet calcification and the short and low coronary ostium. Coronary angiography revealed 50% LAD stent restenosis, diffuse distal LCX disease, and 90% proximal RCA stenosis. A drug-eluting stent was implanted to the right coronary artery. The decision then was made to proceed with TAVI utilizing coronary protection technique during the procedure. TAVR was then undertaken from the right femoral artery through a 14F Edwards arterial sheath. Two 300 mm long Prowater wires were advanced to the LAD and LCX arteries. With rapid ventricular pacing over a long Amplatz extra stiff wire, balloon aortic valvuloplasty using an Edward 4 × 23 mm balloon with simultaneous root aortography to see how the left main flow was performed (Figure 2). There was flow compromise in the left main system to TIMI-2 flow; the wires were deformed and the large piece of calcium in the left cusp moved right over the left main coronary artery (Figure 2). We therefore made decision to place two 3.5 × 12 mm Robel bare-metal stents in the LAD and LCX. An Edward 23 mm SAPIEN 3 valve with rapid ventricular pacing was then deployed followed by deploying the two stents in a kissing fashion, and an excellent result was obtained (Figure 3). Position of the valve and function was confirmed by aortography and transesophageal echocardiography (TEE). The left main coronary flow was excellent. The patient tolerated the procedure well and recovered uneventfully and was discharged 2 days after the procedure without complications. At 30-day follow-up, she has notable improvement of her symptoms and physical activity with NYHA class I from class IV symptoms.

## 3. Discussion

**Figure 4:**
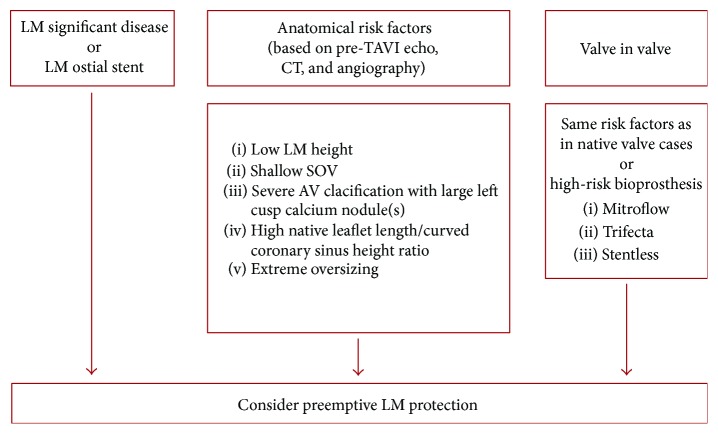
Suggested flow chart for preemptive LM protection based on pre-TAVR evaluation. AV: aortic valve; LM: left main; SOV: sinuses of Valsalva; TAVR: transcatheter aortic valve implantation.

TAVR is an acceptable and successful alternative to surgical aortic valve replacement in high-risk patients. A rare, life-threatening complication of TAVR is a coronary ostial obstruction. Scarce clinical data is available on this important complication as it has been reported in case reports and small case series with an estimated incidence of <1% which carries a high mortality when it occurs (close to 40% at 30 days) [6]. A coronary ostial height cut-off of <10 mm increases the risk of coronary obstruction after TAVI [7, 8]. The LM protection technique should be considered in patients deemed to be at increased risk of LM compromise. It is mostly due to anatomical factors including low coronary ostia and shallow sinuses of Valsalva (SOV) and with valve in valve (VIV) for surgical bioprosthesis. LM protection should be considered in LM height of less than 9 mm, a difference of less than 2 mm between the SOV mean diameter, and the prosthesis diameter or severe aortic valve calcifications with the presence of left cusp large bulky calcium nodule(s). Preprocedural imaging and contingency planning must be utilized as it helps in the early diagnosis and treatment of coronary compromise following valve deployment. This involves preprocedural coronary angiography, EKG-gated, multislice CT angiography study with CT analysis that includes aortic annulus diameter and area, coronary height, SOV diameter, sinotubular junction (STJ) diameter, severity of aortic valve calcification, the presence of aortic valve calcium nodules (>10 mm), prosthesis size/annulus diameter ratio, and prosthesis area/annulus area ratio. Recent report of case series suggested a decision-making flow chart for the preprocedural evaluation of a patient believed to be at increased risk of LM compromise during TAVI (Figure 4) [9].

## 4. Conclusion

The LM protection with preemptive technique is safe and feasible and should be considered in patients deemed to be at increased risk of LM compromise.

## Supplementary Materials

Supplementary Materials Supplementary material contains the slides of the case presentation which took place at CRT meeting in Washington, D.C. on February 19th, 2017.
