# Supplementary material for: Successful Coronary Protection during TAVI in Heavily Calcified Aortic Leaflets in Patient with Short and Low Left Coronary System
Source: Case Rep Cardiol. 2018 May 14;2018:2758170. doi: 10.1155/2018/2758170 (PMC5977029; doi:10.1155/2018/2758170)
Supplement: Supplementary Materials — Supplementary material contains the slides of the case presentation which took place at CRT meeting in Washington, D.C. on February 19th, 2017. [file 2758170.f1.pptx]

## Slide 1
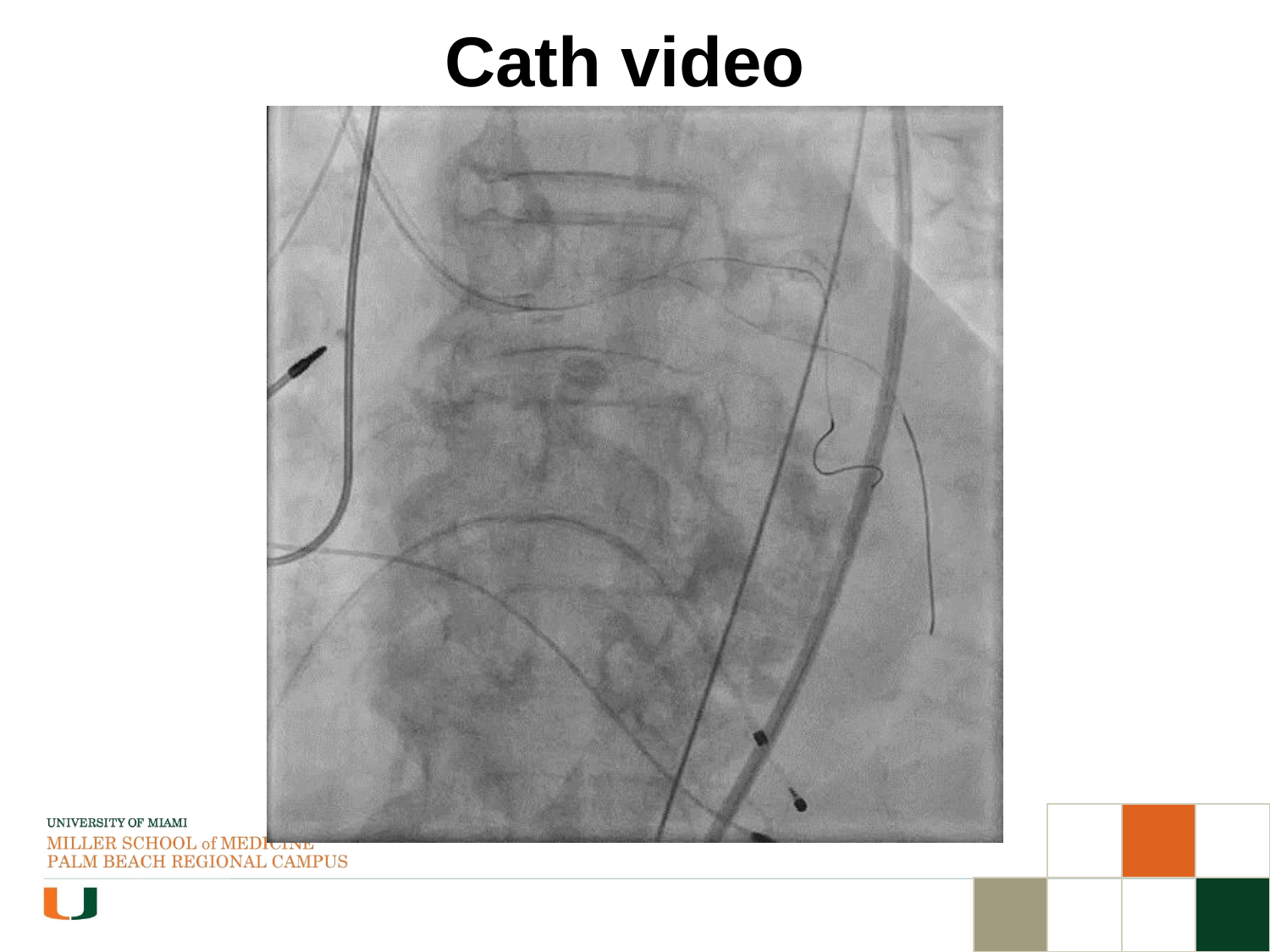

# Cath video

## Slide 2
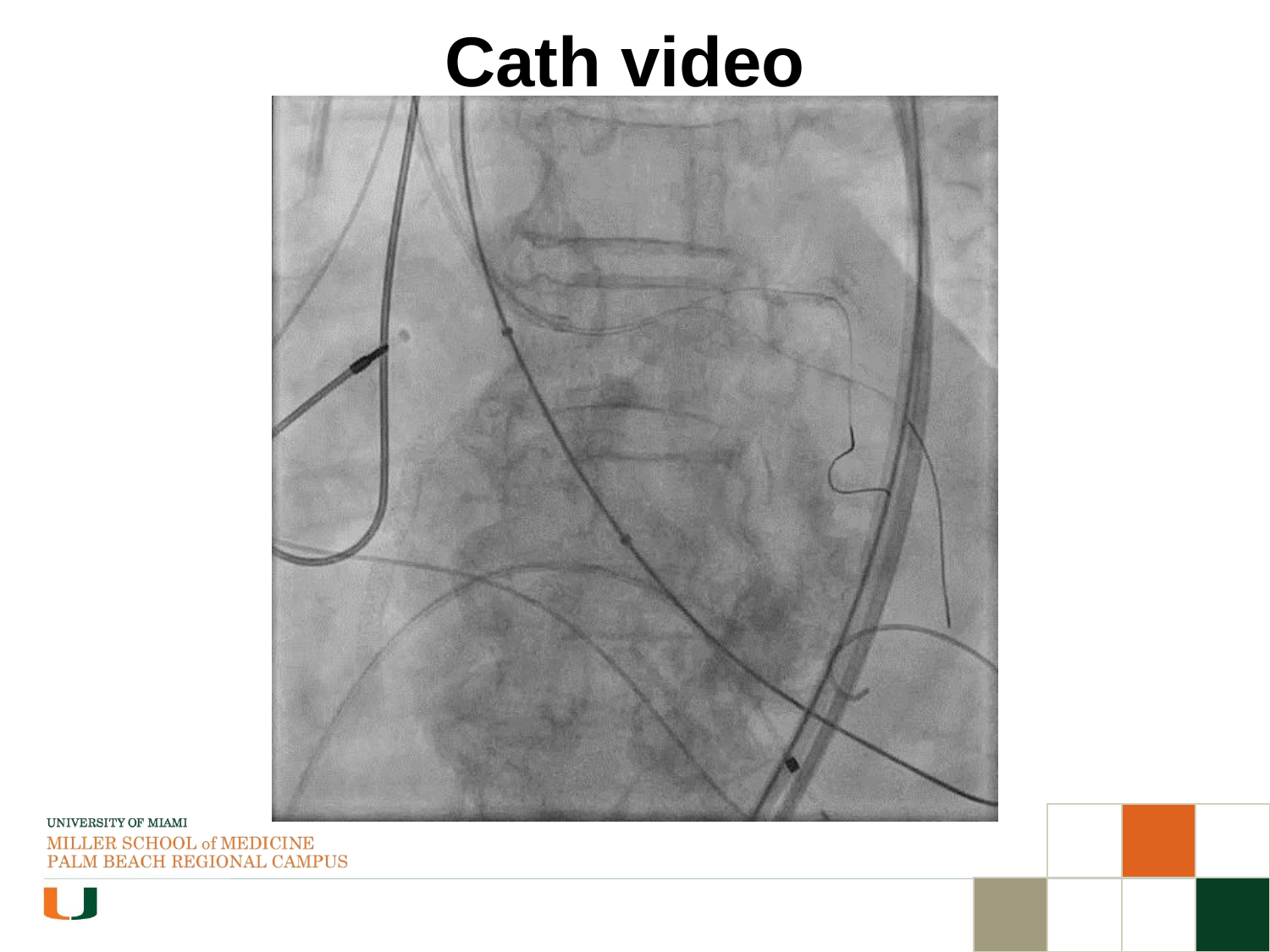

# Cath video

## Slide 3
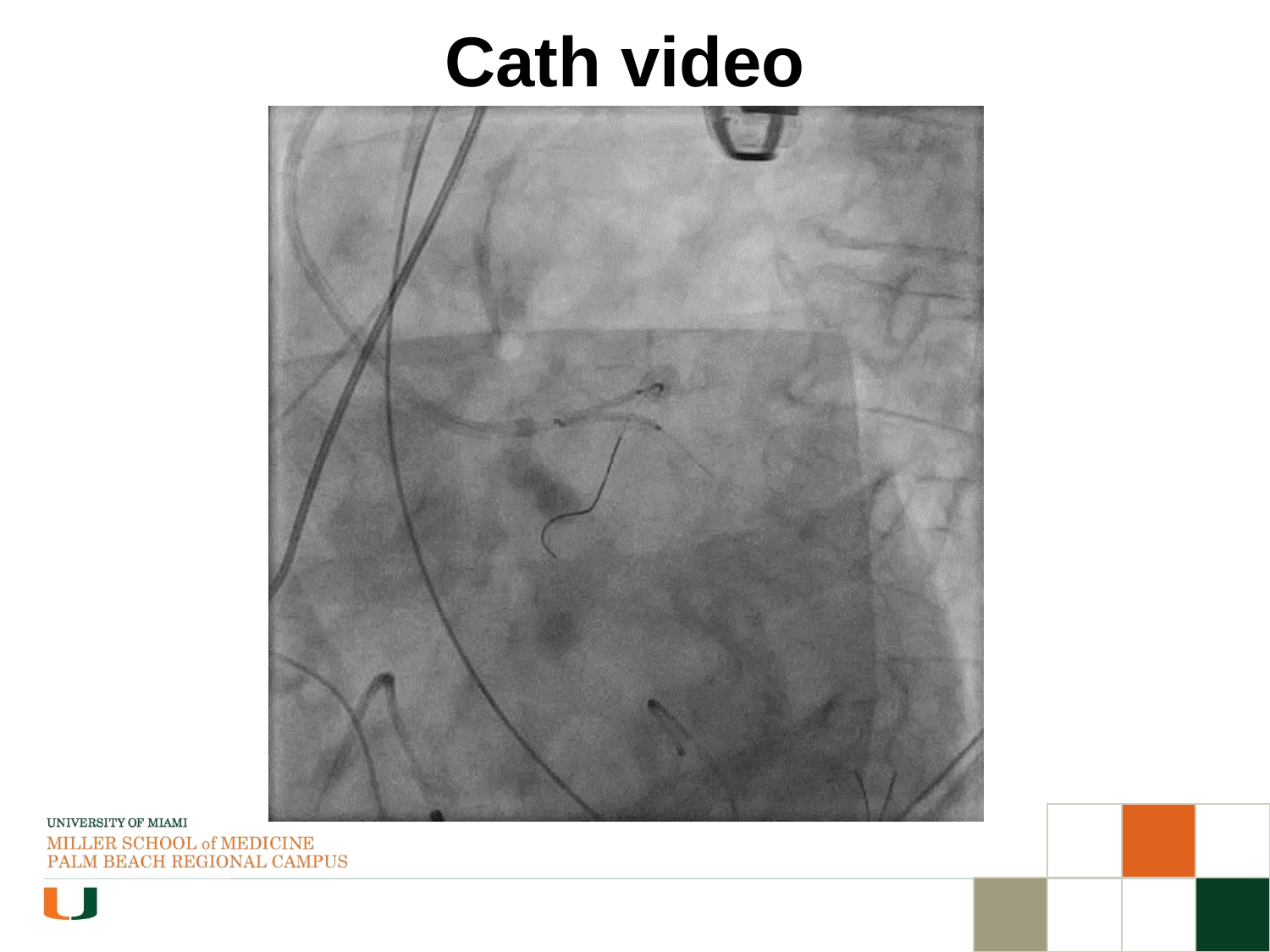

# Cath video

## Slide 4
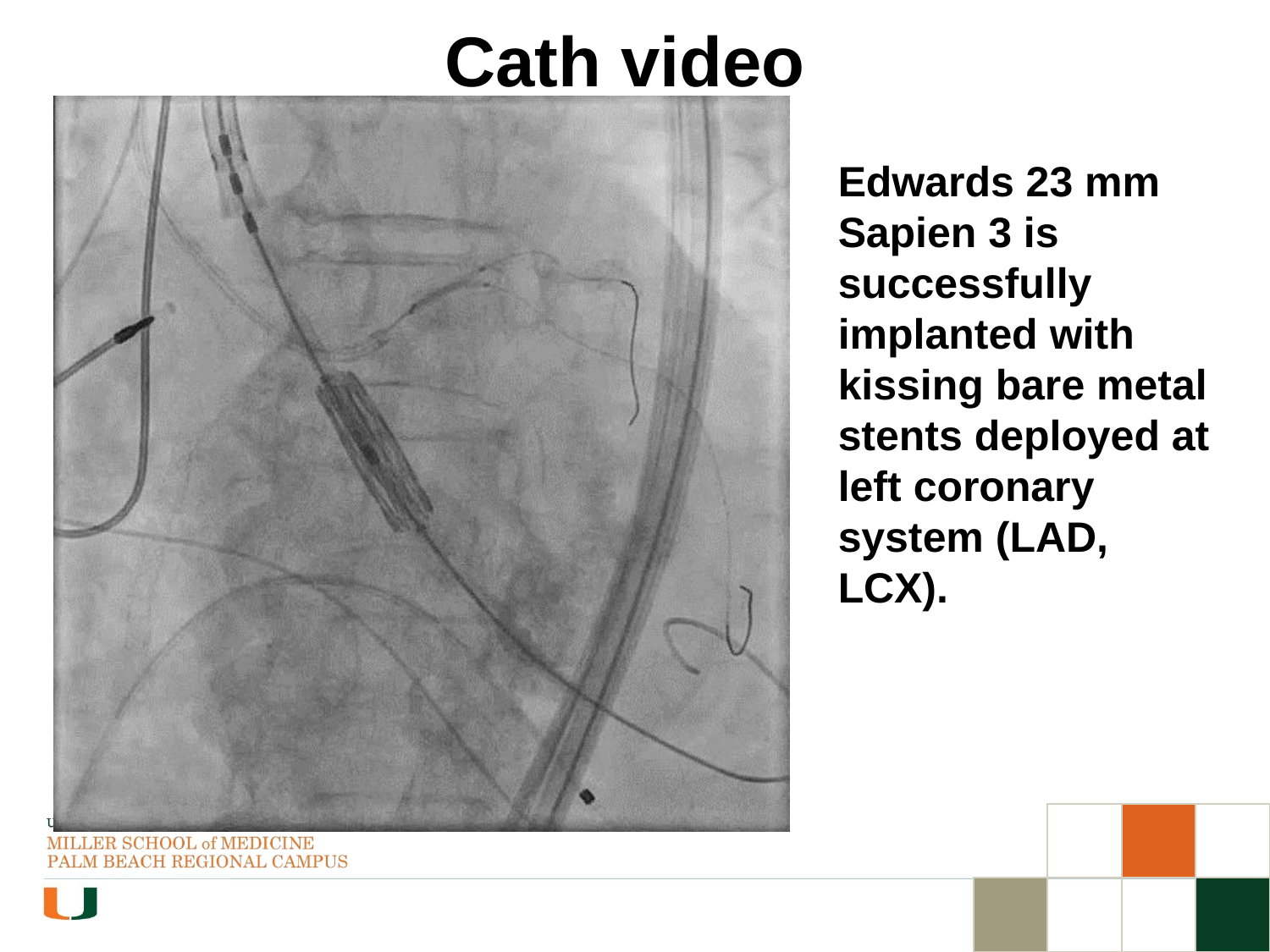

# Cath video
Edwards 23 mm Sapien 3 is successfully implanted with kissing bare metal stents deployed at left coronary system (LAD, LCX).

## Slide 5
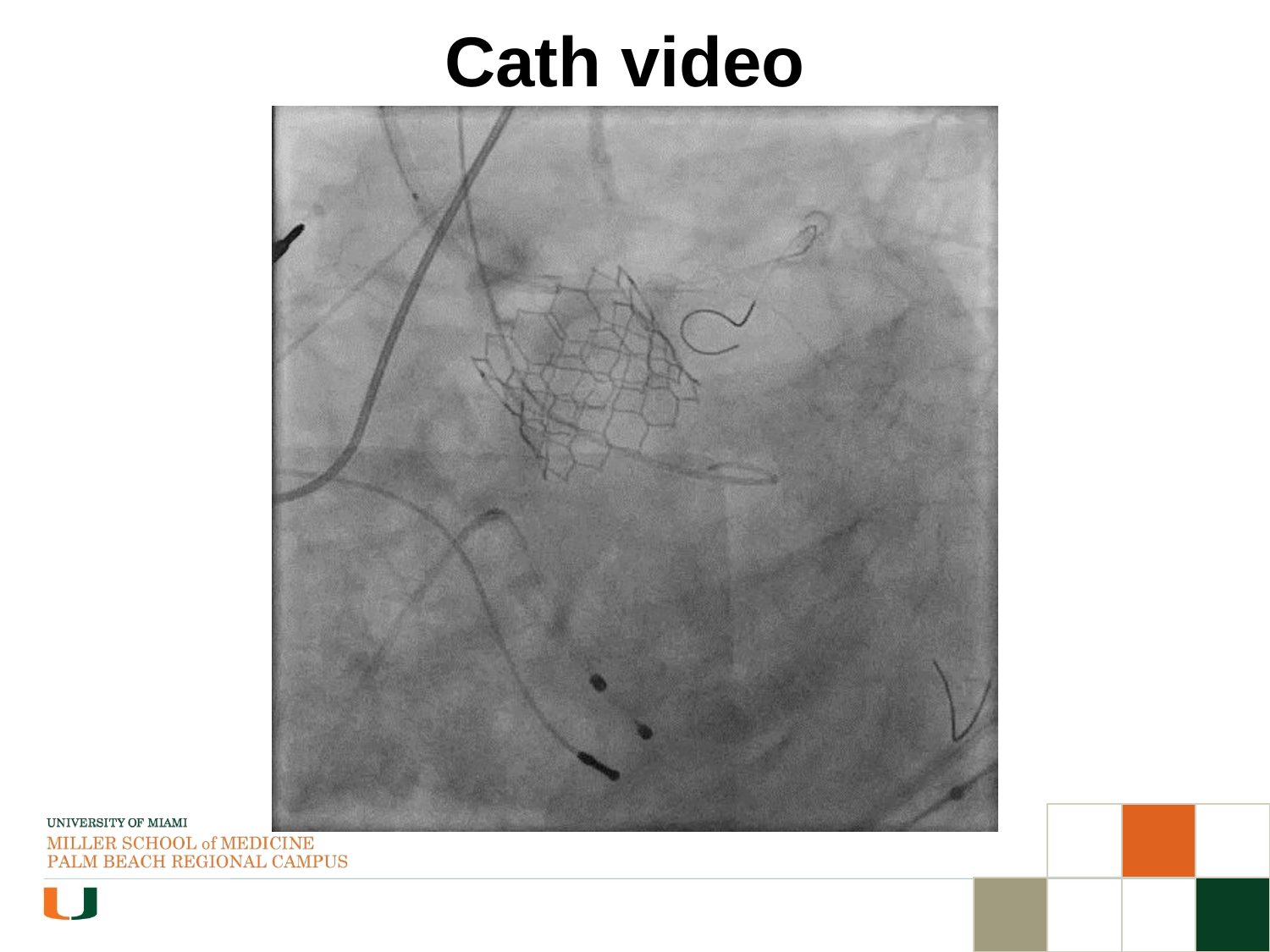

# Cath video
